# Human milk and neurodevelopment in children with very low birth weight: a systematic review

**DOI:** 10.1186/1475-2891-13-94

**Published:** 2014-09-18

**Authors:** Winston Koo, Surinder Tank, Sandra Martin, Runhua Shi

**Affiliations:** Department of Pediatrics, Louisiana State University Health Sciences, 1501 Kings Hwy, Shreveport, LA USA; Shiffman Medical Library, 320 E. Canfield, Detroit, MI USA; Department of Medicine & Feist-Weiller Cancer Center, Louisiana State University Health Sciences, 1501 Kings Hwy, Shreveport, LA USA

**Keywords:** Preterm, Very low birth weight, Milk, Human, Breast milk, Donor milk, Neurodevelopment or cognitive function

## Abstract

Human milk (HM) contains critical nutrients and possibly other neurotrophic factors that could benefit the less developed brain of preterm infants, particularly those with very low birth weight (VLBW). This study aims to systematically review the original studies to determine whether there is a reproducible independent effect of HM feeding on neurodevelopment outcome in preterm VLBW infants. Search of seven databases (PubMed, Cochrane, CINAHL, Embase, Proquest Research Library, Google Scholar, and Web of Science) identified 24 original studies. Each study was evaluated by two authors independently for 8 non-nutritive (study design, target population, a priori power calculation, adjustment for baseline growth status, postnatal complication, other confounders, observer blinding to feeding status, effect size) and 5 nutritive (definition and duration of HM intake, use of HM fortifier, source of HM data, infant formula used) methodology parameters, and consistency and directness of outcome measures. Thirteen reports of preterm infants with wide ranges of birth weights were excluded as none provided sufficient data to delineate the effects of HM feeding on developmental outcome of subjects with VLBW. Eleven reports included only VLBW children and 7 studies were reviewed after elimination of preliminary data from same cohort or lack of appropriate standardized testing or control group. These 7 studies (n = 18 to 704, median 219) were performed at <3 years (3 studies) and at 5 to 11 years (4 studies). Six studies were secondary analysis of data from other studies. Each study met or only partially met 4 to 10 methodological parameters. VLBW children with no neurological impairment fed HM achieved normal or low normal range of test scores. Formula feeding using older formulations was associated with a lower subtest score in 4 studies. There is no randomized clinical trial comparing the neurodevelopment outcome of HM versus formula or minimal HM feeding that included only children with VLBW. The role of HM in the neurodevelopment and cognitive function of VLBW children needs reassessment with high quality studies in the context of current formulations of HM fortifier and preterm formula.

## Introduction

Preterm very low birth weight (VLBW, <1,500 g) infants are at high risk for growth failure and co-morbidities that result in delayed neurodevelopment and academic achievement [1–3]. Early nutrition support is recognized as critical to growth and development and exclusive breastfeeding is universally recommended as beneficial to the health and well-being for all infants [4–6]. However, human milk (HM) alone does not support optimal growth for VLBW infants, so multinutrient fortification, focusing on protein, minerals, vitamins and other nutrients is recommended [4–6]. Preterm VLBW infants are born at a period of significant phase of in utero organ development and are at risk for deficiency of essential nutrients and trophic factors critical to the growth and function of the nervous system. The less developed brain of preterm infants, particularly those with VLBW, theoretically could benefit from feeding maternal milk since it contains critical nutrients such as long chain polyunsaturated fatty acids (LCPUFA) and possibly other neurotrophic factors. This is supported by a review of earlier studies which indicated that HM has greater neurodevelopment benefits than formula for feeding preterm infants [7].

Significant methodological issues arise in the determination of the effect of feeding HM on neurodevelopment outcome. Since it is neither feasible nor ethical to assign breastfeeding randomly, determining the effect of breastfeeding is invariably based on observation. This has challenges particularly in controlling for factors to minimize the risk of bias [8–10]. One quasi-randomized trial in preterm infants of varying birth weights and gestational ages showed significant neurodevelopment benefit for infants fed HM [11]. This and other reports [7], did not adequately control for perinatal and postnatal complications, social and environmental factors that can affect neurodevelopment, and had limited data on the role of HM feeding on neurodevelopment of the group at greatest risk for neurodevelopment impairment, namely children with VLBW. The aim of this study is to systematically review the original studies to determine whether there is a reproducible independent effect of HM feeding on neurodevelopment outcome in preterm VLBW infants.

## Methods

### Identification of articles

A systematic search of the literature was conducted for studies published in English that examined the effect on neurodevelopment and cognitive outcomes from breast milk feeding to VLBW infants. Literature searches of the databases Medline (via PubMed) from 1988, Cochrane Library from 1982, Cumulative Index to Nursing and Allied Health Literature (CINAHL) from 1992, Embase from 1988, Proquest Research Library from1990, Google Scholar from 1994, and Web of Science from 1992 were performed on several occasions with a final search performed on August 11, 2014.

The PubMed search strategy employed a 5 step process using medical subject headings (mh) and related subject/keyword/text word (tw) terms. The first 4 searches were performed independently followed by the 5th search which combined the results from first 4 searches within each database to obtain the articles to be screened for relevance and subsequent review. The first search include infant, premature (mh), OR infant, very low birth weight (mh) OR Very low birth weight (tw) OR extremely low birth weight (tw) OR preterm infants (tw). The second search include breast feeding (mh) OR milk, human (mh) OR breast milk (tw) OR donor milk (tw) OR donor human milk (tw) OR maternal milk (tw). The third search include child development (mh) OR cognition (mh) OR intelligence (mh) OR neurodevelopment (tw) OR cognitive development (tw) OR brain development (tw) OR cognitive outcomes (tw) OR development cognitive (tw). The fourth search include outcome (all fields) OR effect (all fields). The fifth search combined the results from first 4 searches. This procedure was followed for all databases except for some variation in the search terms specific to a database.

For the purpose of this review, “human milk” was defined as breast milk from the mother (own mother’s milk, OMM) or one or more donors (donor milk, DM), whether it was delivered by gavage or a nipple from the bottle or breast. “Neurodevelopment outcome” was defined as the attainment of age-appropriate developmental milestones or specific testing of intelligence or educational achievement. Study outcome limited to behavior/temperament tests or motor ability alone were not considered since their value as the sole predictor of long term neurodevelopment or cognitive function is not well established.

Titles and available abstracts of all studies compiled from the final electronic database search were screened by the investigators to determine eligible studies. Peer reviewed original studies independently assessed the relationship between HM and neurodevelopment outcome were identified. Reports of VLBW children studied as part of a larger cohort of preterm children with greater range of birth weights were included if the data clearly delineated to allow assessment of neurodevelopment effect on VLBW children from HM feeding. Bibliographies from these articles were also searched for additional applicable studies. For each cohort with multiple publications, only the publication with the longest duration of neurodevelopment follow-up was included in this review.

### Evaluation of articles

We evaluated each article following the principles of systematic review [12] and similar to previous reports [8–10, 13] but with modifications pertinent to the VLBW situation. To minimize bias of this systematic review, each study was evaluated independently by two authors (WK and ST) according to a list generated a priori, and the final result was a consensus reached by both authors.

To minimize bias within each study and across studies, each study was reviewed according to a list of non-nutritive and nutritive parameters. The non-nutritive parameters included 1) study design and whether the study’s primary goal was the determination of the effect of breast milk on neurodevelopment or a secondary analysis in a non-breast milk related project, 2) target population, whether VLBW infants were included as part of the preterm population with higher birth weights or were the sole target, 3) predetermined sample size for different feeding groups, 4) whether adjustments were made for baseline differences in other variables such as the presence of intrauterine growth retardation (IUGR), 5) documentation of the extent of postnatal complications that could compromise the neurodevelopment outcome including the extent of intracranial hemorrhage, chronic lung disease, necrotizing enterocolitis, retinopathy of prematurity, severe neurosensory impairment, documented sepsis; how this information was managed and whether infants with serious complications were excluded from the data analysis, 6) control for bias in neurodevelopment and cognitive outcome, namely whether studies were controlled for socioeconomic status, maternal intelligence, and child rearing environment using Child Life Experience [14], Home Observation for Measurement of the Environment [15] or similar assessment tool, 7) whether observers of the outcome were blind to feeding status, 8) whether the study reported an effect size or some other strategy to interpret the clinical impact of the results. Nutritive parameters on the availability of feeding data included 1) definition and 2) duration of HM intake, 3) the type and amount of fortification, 4) source of HM feeding data, and the 5) type of non-HM feeding support. To accommodate the varied ages and circumstances of included children, all data from standardized tests of neurodevelopment or general intelligence were included. All quantitative and statistical data presented were based on each publication without any assumption or modification.

We also assessed the quality of each study [16] according to study design, whether the methodological criteria were met, and consistency and directness of outcome measures.

## Results

**Figure 1:**
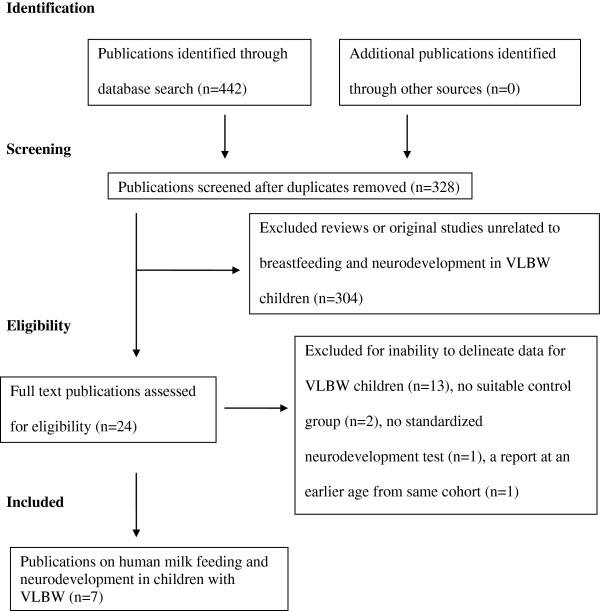
**Search strategy to obtain original studies assessing the effect of feeding human milk on neurodevelopment of children with very low birth weight.**

Figure 1 indicates the number of articles screened and the final number of studies reviewed. A total of 24 reports of original studies that included HM feeding and neurodevelopment outcome in VLBW children were identified. Thirteen reports were excluded as none of these publications provided sufficient data to delineate the effects of HM feeding on developmental outcome of subjects with VLBW [11, 17–28]. Of these 13 publications, six [11, 22–26] were reports of selected subsets (n = 50 to 438) from the same original study populations of 926 subjects. The average birth weights were ~1400 g and 26 to 38% of the subjects were small for gestation (SGA). Two population-based cohorts [17, 28] from the same country included >1400 preterm infants in each cohort. The mean (SD) birth weights of HM fed groups were 1430 (SD 280) g and 1460 (SD 400) g respectively. The birth weights were significantly higher (average 100 g) and as was the mean gestation (average ~0.5 week) than non HM groups. Five other publications [18–21, 27] reported neurodevelopment of subjects with birth weights up to 2000 g (n = 39 to 388). Two of these publications [19, 21] reported on the same cohort.

Eleven reports included only VLBW children [29–39]. Four of these publications were excluded because there was another publication of the same cohort at a younger age [36], no formal standardized neurodevelopment tests were performed [37] and no suitable control group was defined as the study involved supplementation of docosahexaenoic acid and arachidonic acid of HM fed infants [38, 39]. Of the remaining 7 studies of VLBW children, 2 included only children with birth weights <1000 g [34, 35].

Table 1 shows clinical characteristics of 7 studies of developmental outcome associated with HM feeding in VLBW infants. Three studies [30–32] reported the number of SGA infants with birth weight of <10th percentile as surrogate for IUGR. SGA was reported to be as high as 62% in one study [31]. The numbers of children from multiple births also were not well documented.

**Table 1:** **Human milk (HM) and developmental outcome in very low birth weight (VLBW) infants: clinical characteristics**

Reference	Pinelli et al. [29]	Furman et al. [30]	Tanaka et al. [31]	Horwood et al. [32]	Smith et al. [33]	Johnson et al. [34]	Vohr et al. [35]
Birth weight g	<1500	600-1499	<1500	<1500	<1500	<1000	<1000
Gestation: mean (weeks, SD if available)	HM (29, 3) vs. Control (29, 3).	All subjects (27, 2).	HM (28.7, 3.2); Control (30.7, 1.6).	*	HM (28.7, 2.4) vs. Control (27.8, 2.5).	HM <26 vs. Control (term birth).	HM 26.7 vs. Control 26.2.
Range of HM intake	>80% vs. <80%	>50 to 0 mL/kg/d	>80% vs. <80%		Breastfeeding at discharge vs. no HM at any stage	VLBW infants: any breast milk vs. none during hospitalization. Control: regardless of HM intake	>80% vs. 0
% small for gestation	NS	8	HM 40%; “Control” 62%	*	NS	NS	NS
% singleton	100	100	NS	*	61.5	NS	NS
Year/s of birth	NS	Jan 1997-Feb 1999	1999-2000	1986	1991-1993	Mar-Dec 1995	Oct 99-Jun 2001

NS – not stated or specified.

*Small for gestation and multiple births entered into analysis but no raw data presented.

Table 2 shows details of non-nutritive methodological parameters. All were observational studies dependent on maternal choice whether to provide breast milk with varying amount of OMM provided to their VLBW infant. In 6 of 7 studies, the effects of HM feeding on neurodevelopment were extracted via secondary analysis of data from other projects. The design of the primary studies was epidemiologic observational with 2 interventional studies: one on structured counseling to promote breastfeeding [29] and the other on glutamine supplementation in parenteral nutrition [35]. Three studies [31, 33, 35] included only subsets of the study population from primary study. Exclusion criteria were generally clearly defined although varied among different studies. Three studies [29, 30, 35] included children assessed at younger than 3 years and 4 studies included children from 5 to 11 years [31–34]. Sample sizes varied from 18 to 704 (median 219) children and none stated a priori power calculation to measure the effect of breast milk. The attrition rate of the subjects assessed tends to increase with increased duration of follow-up. Blinding of the observers to the feeding status of the subjects was reported in 3 studies [29, 30, 34].

**Table 2:** **Human milk (HM) and developmental outcome in very low birth weight (VLBW) infants: non-nutritive methodological parameters**

Reference	Pinelli et al. [29]	Furman et al. [30]	Tanaka et al. [31]	Horwood et al. [32]	Smith et al. [33]	Johnson et al. [34]	Vohr et al. [35]
Primary study for the subjects	Structured breast feeding counseling to sustain lactation	Self selected provision of breast milk vs. preterm formula	RBC DHA in breastfed VLBW infants at 4 weeks	Retinopathy of prematurity	Epidemiology of brain injuries in VLBW infants and Epidemiologic study of multiple births	Early predictors of education attainment at 11 y	Parenteral glutamine trial
Subject source	One center, Canada	One center, USA	One center, Japan	All VLBW births, New Zealand	5 centers, USA	All extremely preterm births in United Kingdom and Ireland	12 of 15 NICHD NRN sites, USA
Exclusions	Multiple birth, severe congenital, surgical, chromosome abnormality, non-English speaking parents	Positive drug screen, major congenital anomalies, intrauterine infection, overwhelming maternal social concerns	Cerebral palsy, no RBC DHA data, severe chronic lung disease, minor anomalies, hearing problem	Sensorineural deficit and no breast milk data	NS	None	Unable to test including those with sensorineural deficit
Assessment age	6 and 12 m corrected	20 m corrected	5y	91 m	6 to 8y	Median 10y 11 m	30 m corrected
Sample size (assessed/eligible)*	138/148	98/119	18/26	298/338	439/770	219/307; and 153 term “Control”	704/939
Observers blinded to feeding protocol	Yes	Yes	NS	NS	NS	Yes	NS
Neurodevelopment tests	BSID II: MDI and PDI	BSID II: MDI and PDI	KABC	WISC-R: verbal and performance IQ	CELF; CCVL; KABC; PPVT; WRAVMA	KABC; TAAS; WIAT-II	BSID-II
Effect size for human milk†	See below	See below	See below	See below	See below	See below	See below

CI – confidence interval; NICHD NRN – National Institute of Child Health and Human Development Neonatal Research Network; NS – not stated or specified or not significant; RBC DHS red blood cell - Docosahexaenoic acid; SD – standard deviation.

Neurodevelopment tests: BSID II Bayley Scales of Infant Development (2nd edition), MDI Mental Developmental Index, PDI Psychomotor Developmental Index; CCVL California Children’s Verbal Learning Test; CELF Clinical Evaluation of Language Fundamentals 3rd ed; KABC - Kaufman Assessment Battery for Children; PPVT - Peabody picture vocabulary test 3rd ed; TAAS Teachers Academic Attainment Scale; WIAT-II Wechsler Individual Achievement Test – II; WISC-R - Revised Wechsler intelligence scale for children; WRAVMA - Wide Range Assessment of Visual Motor Abilities.

*None had stated a priori power calculation to measure the neurodevelopment effect of HM. For Smith et al. [33], total sample size included 4 gestation matched VLBW controls for each VLBW subject with abnormal cranial ultrasound. For Johnson et al. [34], control subjects included one subject selected randomly from 3 classmates born at term with same sex and ethnicity.

†Effect size for human milk (maximum amount or as specified) vs none or limited human milk. Mean (SD or CI if available) scores after adjustment for covariates and confounders:

Pinelli et al. [29]. Dichotomous groups based on 80% HM intake as cut point: No significant difference in MDI 92 (15) vs. 91 (12) or PDI 78 (15, SD) vs. 77 (14).

Furman et al. [30]. No significant difference in MDI 85 +/−21 vs. 80 +/−16 or PDI 76 +/−16 vs. 80 +/−16.

Tanaka et al. [31]. Dichotomous groups based on 80% HM intake as cut point: higher raw score for sequential 106.7 (14.5) vs. 94.7 (11.6) but not simultaneous or composite mental processing. No adjusted scores.

Horwood et al. [32]. Higher mean verbal IQ (102.1 vs 96.1 p < 0.05) and performance IQ (103.3 vs. 99.6, p > 0.15) after >8 m HM.

Smith et al. [33]. Significantly different in visual motor skills only: WRAVMA drawing 97.7 (14.6) vs. 90.6 (13.5), 95% CI = 1.0-9.2; and K-ABC triangle completion 10.6 (3.0) vs. 9.1 (2.5), 95% CI = 0.1-1.7. No HM effect from increased HM duration (>4 m, in infants with abnormal cranial ultrasound or <28 weeks gestation).

Johnson et al. [34]. Regardless of HM intake, significantly lower composite scores in extremely preterm children without serious neurosensory or cognitive impairment for reading 91 (13.4) vs. 98.7 (11.5); for mathematics 84.0 (15.6) vs. 98.8 (14.8). Data analyzed for children with and without serious functional or cognitive impairment while attending mainstream or special schools. Multivariate model show breast milk is one of the independent predictors of reading scores but not mathematic scores at 11 years. Other independent predictors included BSID-II MDI, and head circumference at 30 m, and perinatal and social factors. All independent predictors accounted for 31% of the variance for reading scores at 11 years.

Vohr et al. [35]. MDI and PDI in the highest 3 quintiles of human milk groups were higher (p <0.05) than no human milk group. Mean values for highest vs. no human milk for MDI 89.7 vs. 76.5, and for PDI 90.2 vs 78.4. HM intake as a continuous measure show that each 10 mL/kg/d increase in human milk ingestion, the estimated increase of 0.59 points in MDI and 0.56 points in PDI.

In all studies, VLBW children without neurological impairment fed HM achieved normal or low normal scores on standardized tests of neurodevelopment or cognitive function. Thus any advantage of HM feeding is due to the lower scores of formula-fed infants. Two studies [29, 31] used dichotomous grouping with 80% of the intake of HM as a cutoff point. For the group that received the most HM, one of these two studies reported a higher raw score in one subtest of neurodevelopment at 5 years of age but no adjustments were made to account for confounders [31]. The other study showed no significant difference in adjusted scores [29]. One study at 30 months corrected age showed HM feeding during the hospitalization resulted in mean Mental Developmental Index (MDI) and Psychomotor Developmental Index (PDI) scores on the Bayley Scales of Infant Development (BSID) in the low normal range; those fed infant formula have significantly lower scores [35]. Two of the 3 studies with neurodevelopment assessment at 6 to 11 years showed that HM feeding at highest volume was associated with intelligence test scores in the normal range. The formula fed group showed a significantly lower score in verbal intelligence quotient (IQ) in one study [32] and in visual motor skill subtest in another study [33]. The third study [34] reported that children born extremely preterm without major neurological impairment showed significantly poorer academic attainment on reading and mathematics than their term peers; HM feeding positively affected reading, but not mathematics attainment, at 11 years. In the same study, breast milk consumption together with perinatal and neonatal complications and socio-economic status accounted for 29% of the variance in reading attainment at 11 years.

Table 3 shows every study obtained some data on perinatal, postnatal, social and environmental factors. However, the extent of the details varied greatly. The statistical handling of the numerous variables also varied among studies and only one study adjusted for the SGA status.

**Table 3:** Human milk (HM) and developmental outcome in very low birth weight (VLBW) infants: perinatal, postnatal, social and environmental data*

Reference	Perinatal/postnatal factors	Social and environmental factors
Pinelli et al. [29]	Type of delivery	Maternal and paternal age, education and occupation, 1 or 2 parent home, social classes I-V (Hollingshead index)
Furman et al. [30]	Delivery at perinatal center, antenatal steroid and cesarean section. Apnea, sepsis, jaundice, necrotizing enterocolitis, chronic lung disease, cranial ultrasound abnormalities.	Maternal education and ethnicity, and marital status
Tanaka et al. [31]	Chronic lung disease, cranial ultrasound, necrotizing entercolitis. Intrauterine growth retardation	Maternal age and education
Horwood et al. [32]	Sex, multiple births, birth weight, gestational age, intrauterine growth retardation, 5 min Apgar score	Maternal age, education and smoking, 1 or 2 parents, family income, child ethnicity, birth order
Smith et al. [33]	Length of hospital stay	Maternal age, verbal ability, education, cigarette smoking and marital status, Home observation for measurement of the environment inventory – short version, annual household income, gender, parity
Johnson et al. [34]	Birth weight, gestation, antenatal steroid, premature rupture of membranes, vaginal breech delivery, chorioamnionitis, admission temperature <35°C, CRIB score, abnormal last cranial ultrasound, necrotizing entercolitis, postnatal steroid, duration of NICU admission. Neurodevelopmental assessment results at 30 m and 6 y	Socioeconomic (UK National Statistics Socio-Economic classification), maternal age, race and highest education.
Vohr et al. [35]	Gestation, gender, sepsis, intraventricular hemorrhage grade 3 to 4, periventricular leukomalacia, oxygen need at 36 weeks, necrotizing enterocolitis, and weight <10th percentile at 18 months.	Maternal age and education, marital and health insurance status, race, and income.

*The variables entered into the final model to determine the independent effect of HM feeding were varied and not always fully described. Some investigators [30] used composite scores to minimize the number of variables entered into data analysis and no specific modeling was performed by other investigators [31].

Table 4 shows the nutritive parameters assessed in each study. Details on the extent and duration of HM feeding varied. The source of the feeding data was referenced in 4 studies. Those with short-term follow-up generally had sufficient details to allow categorization of the amount of HM intake. Long-term follow-up studies generally relied on maternal recall, and the amount of HM intake was not quantified [31–34]. Only one study reported use of health clinic record [32] as an additional measure to minimize recall bias. No study reported the use of donor milk. Use of HM fortifier was reported in 2 studies. Preterm infant formula usage was reported in 3 studies but the type of infant formula used was not documented in the other 4 studies. In 6 of 7 studies, the control group used as a comparison with HM-fed group was from the same cohort of VLBW infants who were fed exclusively infant formula or whose daily feedings consisted of up to 80% HM. Only one study included children born at term and matched for sex and ethnicity and attending the same mainstream school or special education facility.

**Table 4:** **Human milk (HM) and developmental outcome in very low birth weight (VLBW) infants: nutritive parameters**

Reference	Pinelli et al. [29]	Furman et al. [30]	Tanaka et al. [31]	Horwood et al. [32]	Smith et al. [33]	Johnson et al. [34]	Vohr et al. [35]
HM feeding definition. None specified whether donor milk was used.	Maternal milk intake as % of total fluid intake and by duration	Maternal milk at 0, 1–24, 25–49, >50 mL/kg/d	Maternal milk	Any maternal milk from birth	Expressed maternal milk without or with progression to direct breastfeeding	Any breast milk	Maternal milk intake by quintiles
HM feeding duration	Continuous measures till 12 m corrected	Up to 4w	HM group 72 +/− 45.2 (SD)d, Formula group received HM for 59 +/−32.1d	None, <4 m, 4-7 m, 8 + m	<1w, 1-4w, 1-3 m, 4–6 m, >6 m	Neonatal period	Up to 120d.
HM fortification	Milk based powder if intake <180-200 mL/kg/d (21% of infants)	Milk based powder or concentrated PTF	NS	NS	NS	NS	NS
HM feeding data source	Maternal questionnaires, 24 h expressed milk volume, test weighing one feeding each 3 m	*	*	Maternal recall and child health record	Maternal recall	*	Database from hospital records
Infant formula data	20 exclusively PTF	PTF	NS	NS, n = 76	NS	NS	PTF, 180 (23%) exclusively FF

PTF - Preterm formula in hospital, FF - formula fed, NS - not stated or specified.

*Not stated and presumably were obtained from review of hospital record.

All but one study were secondary analyses of data from primary studies that may have an independent effect on neurodevelopment. The effect of HM on neurodevelopment was based on observation of selected cohorts. The quality and the risk of bias, as determined by the extent to which the methodological parameters are met, were varied among different studies (Table 5). Of the 8 non-nutritive parameters, each study has at least 4 parameters that were either not met or only partially met with the use of surrogate markers. Of the 5 nutritive parameters, one study did not meet one parameter while the other studies did not meet 2 or 3 parameters (Table 5). Lack of documentation for a methodological parameter or the use of surrogate markers negatively affected the quality of many studies. Consistency of the effect is variable with the advantage of feeding HM in adjusted neurodevelopment or educational attainment test scores in 4 of the 7 reports [32–35]. Three of these studies [32–34] of VLBW children at 6 to 11 years of age showed an advantage from HM with selected subtests rather than overall test scores. Dose effect of HM intake was reported with 2 studies [32, 35]. Directness of the outcome was supported with the use of age-appropriate standardized tests but suffered from secondary analysis of other studies, incomplete sampling, poor selection of control group, and use of surrogate markers.

**Table 5:** **Studies of human milk (HM) feeding and developmental outcome in very low birth weight (VLBW) infants: meeting criteria for methodological quality**

Reference	Pinelli et al. [29]	Furman et al. [30]	Tanaka et al. [31]	Horwood et al. [32]	Smith et al. [33]	Johnson et al. [34]	Vohr et al. [35]
As primary outcome of original study*	-	+	-	-	-	-	-
VLBW only	+	+	+	+	+	+†	+†
A priori power calculation	-	-	-	-	-	-	-
Baseline adjustment for SGA	-	-	-	+	-	-	-
Postnatal complication	-	+	-	+	-	+	+
Maternal intelligence	+/−	+/−	+/−	+/−	+/−	+/−	+/−
Social class or Socioeconomic status	+	-	-	+/−	+/−	+	+/−
Child rearing environment	+/−	+/−	-	+/−	+	-	+/−
Observers blinded to feeding protocol	+	+	-	-	-	+	-
Effect size after adjustment	+	+	NA	+^‡^	+^‡^	+^‡^	+^‡^
Human milk definition	+	+	+	+	+	+	+
Human milk duration	+	+	+	+	+	+	+
Human milk fortification	+	+	-	-	-	-	-
Human milk feeding data source	+	-	-	+	+	-	+
Formula type	+	+	-	-	-	-	+

SGA = Small for gestational age, NA = not available.

+Met methodological criterion.

-Did not meet methodological criterion; or not stated or not specified in the publication.

+/−Use surrogates such as income for socioeconomic status, maternal education for maternal intelligence, marital status or one or two parent family for child rearing environment.

*All studies were observational and most were secondary analysis of study cohort from other studies.

†Only children with birth weights <1000 g.

^‡^Limited consistency of neurodevelopment outcome. Human milk showed variable advantage in some test scores [32–35].

## Discussion

For infants born at term, the benefits of HM on neurodevelopment and cognitive function may be limited according to reports on studies that adequately controlled for maternal intelligence and other social and environmental factors [8–10]. HM may provide greater benefit for the preterm infants when there is an added need for specific nutrients and trophic effects. Meta-analysis of earlier studies with larger preterm infants supported this assumption [7]. However, the meta-analysis did not attempt to evaluate each study’s methods or interpret results on the basis of the quality of the investigation. As a result, the pooled effect estimates obtained reflect the average of a heterogeneous group of studies.

Our systematic review on the independent effect of HM feeding on neurodevelopment outcome, taking into account the additional confounders unique to VLBW children, provided a better understanding of the strengths and limitations of each study. It appears that significant limitations exist with each study. These limitations may involve study design or the quality of the study in the fulfillment of non-nutritional and nutritional methodological criteria, which can affect the applicability of the outcome data. The inconsistent effect on neurodevelopment test scores and variable advantage in different subtest scores when assessed at school ages also contributed to the difficulty in interpreting the HM effect on neurodevelopment outcome.

Majority of publications determining the effect of breast milk feeding on neurodevelopment in VLBW children is based on observational data from other studies. The source of VLBW population for example those with postnatal complications [32, 33]; and intervention performed in the original studies, such as counseling to improve breastfeeding [29] or glutamine supplementation in parenteral nutrition [35] potentially may influence the outcome measures by indirect means. Furthermore, secondary analysis of data generates more questions for hypothesis testing rather than providing a definitive cause and effect of HM feeding.

Quality of the studies, as indicated by adherence to methodological criteria that minimizes risks for bias, is generally low. Numerous factors other than nutritional intake have been identified as confounding variables in relation to child development [40] and may have origin even before birth. Many preterm infants experienced variable adverse growth in utero but not all studies reported the rate of IUGR or SGA. In preterm infants, SGA is an independent predictor of severe cognitive deficit [17]. For extremely preterm infants with VLBW, SGA as an indicator of IUGR has an odds ratio of 3.91 for increased risk of death or neurodevelopment impairment [41]. Some studies of children born preterm have reported IUGR or SGA rates from 34% [26] to as high as 60% [31], and not all studies reported whether or how the data analysis accounted for IUGR or SGA. Extra uterine growth retardation also occurs frequently in VLBW infants and may be another marker for neurodevelopment delay [42]. A small head circumference at 8 months corrected age is an independent marker of neurodevelopment and cognitive impairment, independent of the type of feeding [43, 44]. Multiple births are at risk for preterm delivery and discrepant in utero growth resulting in VLBW and IUGR, and discordant neurodevelopment outcome has been reported for VLBW twins [45]. Not all studies have accounted for multiple births and some studies have restricted the study population to singletons [29, 30]. In addition, the effect on breast milk production in mothers with both twins admitted to a neonatal intensive care unit is not well-defined.

For extremely preterm infants, a difference in 100 g in birth weight or one week of gestation can have major impact on perinatal and postnatal complications [46] that can directly or indirectly impact neurodevelopment outcome and confound the effects of HM. Thus it is important not to generalize the neurodevelopment effect of HM from preterm infants with higher birth weight and gestation, since they have a relatively longer period of development in utero and less serious postnatal complications. The earlier reports included large numbers of preterm children with higher birth weights than VLBW which could reflect better intrauterine growth at the same gestation or had IUGR at a more advanced gestation [11, 17–28]. Interaction between gender and diet has been reported in some studies, with males showing more benefit from nutritional intervention [11, 26, 34]. One study of children with birth weight <1000 g reported that being male has a small negative predictive effect on reading but not mathematics attainment at 11 years [34].

The age at follow-up varies, although few studies [32–34] assessed cognitive scores at school ages which are considered as much better predictors of adult scores. One report of significant positive effect of HM on neurodevelopment at 30 months corrected age was generated from secondary analysis of a subpopulation from another project [35]. In the same cohort, there was a significant increase in MDI scores by 2.7 points in the HM group and a trend to lower PDI scores by 2.3 points in the non-HM group since an earlier assessment at 18 months [36]. This drift in test scores could bias the outcome that showed HM group has higher MDI and PDI scores at 30 months [35]. The advantage associated with HM feeding appears to diminish with older children, as the improvement in test scores is limited to selected and different subtests [32–34]. It is possible that the effect of HM may be less important as genetic and environmental factors play bigger roles at school age. In some cohorts with follow up at older ages, the validity of mother’s milk effect on IQ at adolescence is questionable since the data is based on <10% of the subjects from the original cohort [22].

Age-appropriate developmental or cognitive tests standardized to normal age matched children allow the use of a control group from the same VLBW cohort [29–33, 35]. Only one study used selected classmates in the same educational setting and born at term with same sex and ethnicity and tested during the same period to minimize any secular drift in test scores over time [34]. Selection of a control group based on the volume of human milk ingested heavily influences the outcome. Any HM effect on neurodevelopment may be difficult to detect when the data analysis is dichotomized using a large volume of HM consumption as a cutoff point [29, 31]. Not all reports indicated whether the testers were blind to feeding status thus contributing to the risk for bias.

The extensive numbers of potential confounders of neurodevelopment and variable exclusion criteria based on the type and extent of postnatal complications support the need for appropriate statistical modeling and large sample size to provide meaningful interpretation of the neurodevelopment effect of feeding HM. One study employed a composite neonatal risk score and a composite socioeconomic score to minimize the number of independent variables and to avoid multi-colinearity of variables in statistical modeling [30]. However, there is no uniform approach to statistical modeling and none of the studies had stated a priori power calculation to measure the neurodevelopment effect of HM.

Both breastfeeding and neurodevelopment outcome are confounded by maternal intelligence, social and socioeconomic status, and child rearing environment, and possibly from intangible psychobiology of maternal behavior and mother-infant relationship [10, 47, 48]. Mothers elected to provide HM and breastfeeding are often highly motivated and possibly more health conscious and more likely to stimulate their infants thus contributing to self-selection bias. None of the studies reviewed have formal assessment of maternal intelligence and few studies specifically assessed the other confounders. The use of surrogate for these critical independent determinants of neurodevelopment outcome limits the validity of HM effect.

It is important to recognize that VLBW children with no neurological impairment and fed HM achieved normal or low normal scores on standardized tests of neurodevelopment or cognitive function. The VLBW children fed a lower amount of HM or fed infant formula have lower test scores. One should consider feeding HM as protective rather than providing an added advantage to neurodevelopment.

Nutritive factors are important in the evaluation of the role of HM in neurodevelopment and cognitive function of VLBW children. In a cohort of children born preterm and including those with VLBW enrolled in a quasi-randomized study of supplementing OMM with DM or infant formulas, preliminary data at 7.5 to 8 years from the first 300 children out of 926 subjects showed that the children who received OMM had IQ scores in the normal range. However, those who exclusively received formula have overall test scores that were 8.3 points lower [11]. In a subsequent report of 377 subjects from several subsets in the same cohort of infants, those who received infant formula (nutrient enriched versus regular formula), and either exclusively or as a supplement to OMM, experienced a beneficial effect to neurodevelopment that appeared primarily to be related to the use of the nutrient enriched formula [26]. Unfortunately, no quantifiable data specific to VLBW children was presented in either report.

In the studies reviewed, the contents of nutrients in infant formulas and HM fortifiers are in much lower quantity and lack additional nutrients such as LCPUFA when compared to the current formulations. If better nutrient profile is critical to neurodevelopment, then it is possible that all VLBW infants could benefit with the use of newer and better fortified formulas and HM fortifiers.

The timing, volume and duration of HM consumed could be important for neurodevelopment. Significant advantage in neurodevelopment effect appears to occur even after a brief period of consumption of OMM during initial hospitalization as >88% of infants in one study [11] and 77% of infants in another study [35] received no HM by the time of hospital discharge. Unfortunately, these details are extremely limited in long-term follow-up studies.

A dose effect of OMM also may be present. Preterm infants whose mothers intended to breastfeed but could not provide any breast milk, performed at the level of exclusively formula fed children on cognitive testing at 7.5 to 8 years [11], and a dose effect was also demonstrated when the analysis was performed with HM intake as a continuum [35]. One long-term VLBW population cohort at 7 to 8 years of age showed a significant benefit of prolonged breastfeeding and reached a mean of 6 points advantage in verbal IQ after receiving OMM for 8 months or more [32]. However, no additional benefit beyond 4 months of breastfeeding was reported in another study of VLBW children at 6 years [33]. A modest independent beneficial effect of feeding OMM during the neonatal period on higher reading but not mathematics attainment at 11 years was also reported [34].

It is important to determine whether OMM or DM was used in the assessment of HM’s effect on neurodevelopment. Fresh OMM contains many components that may provide trophic actions which can directly or indirectly influence the growth and development of the nervous system but are inactivated or destroyed during processing of the DM [6, 49]. The use of DM alone or as supplement to OMM resulted in poorer growth and neurodevelopment [23]. It appears that VLBW children in all studies reviewed were provided with OMM; none reported that any DM was used. The use of HM fortifiers was reported in two studies [29, 30]. In another study, the use of human milk fortifier was not specified but was likely provided since the cohorts were born in the era when HM fortification was the standard of care [35].

It is possible that space limitation imposed by the journals may have precluded detail description by the investigators, although it is unlikely to eliminate all significant limitations that exist with each study. Limitations to our study included at least the following: we evaluated original peer reviewed studies only in English and did not pursue details from published abstracts or the authors. However, abstracts have not undergone the same rigor in review process as the full publication and are unlikely to have sufficient data to allow meaningful systematic review of the data. Information from the authors is unlikely to resolve the many methodological concerns in the studies reviewed, and additional information would be unlikely to alter the overall conclusions. Our study also excluded 13 reports [11, 17–28] because of insufficient data to delineate the effect of HM feeding on neurodevelopment outcome in children with VLBW. The data from these reports included many children with higher birth weights and thus at lower risk of neurodevelopment deficit than VLBW children. Furthermore, reports based on subsets of original cohort make it difficult to interpret the significance of the finding in the context of the whole population and should be considered as hypothesis generating rather than definitive data on the neurodevelopment benefit of HM.

In addition, the two population observational studies [17, 28] were confounded by the HM fed cohorts having significantly greater birth weights and gestations compared to non HM group. In any case, the neurodevelopment scores in those fed predominantly OMM [11, 17–28] are generally within the normal ranges and consistent with reports that include only children with VLBW [29–35].

## Conclusions

There is no randomized clinical trial comparing the neurodevelopment outcome of HM versus formula or minimal HM feeding that included only children with VLBW. Studies to date have significant methodological limitations although limited data suggest a possible protective effect on neurodevelopment from feeding OMM for a short period after birth and a possible dose effect on the volume and duration of feeding OMM. If better overall nutrient profile is more important for optimal neurodevelopment, it is theoretically possible that the use of current formulations of HM fortifier and preterm infant formulas could improve the neurodevelopment outcome of all VLBW children. Thus, the role of maternal milk in neurodevelopment and cognitive function of VLBW infants needs to be reassessed with high quality studies in the context of current formulations of HM fortifier and preterm formula. With increasing use of DM to the exclusion of preterm formula in NICU, a separate assessment of the role of DM in neurodevelopment is needed in view of the numerous differences between OMM and DM.

## Abbreviations

DM: Donor milk
HM: Human milk
IQ: Intelligence quotient
IUGR: Intrauterine growth retardation
LCPUFA: Long chain polyunsaturated fatty acids
OMM: Own mother’s milk
SGA: Small for gestation
VLBW: Very low birth weight.
